# Polyetherimide hollow fiber membranes for CO_2_ absorption and stripping in membrane contactor application

**DOI:** 10.1039/c7ra12045a

**Published:** 2018-01-17

**Authors:** R. Naim, A. F. Ismail, T. Matsuura, I. A. Rudaini, S. Abdullah

**Affiliations:** Faculty of Chemical and Natural Resources Engineering, Universiti Malaysia Pahang Lebuhraya Tun Razak 26300 Kuantan Pahang Malaysia rosmawati@ump.edu.my jem4028@gmail.com +60 95492889 +60 95492876; Advanced Membrane Technology Research Centre (AMTEC), Universiti Teknologi Malaysia 81310 Skudai Johor Malaysia afauzi@utm.my fauzi.ismail@gmail.com +60 75581463 +60 755535592; Department of Chemical and Biological Engineering, University of Ottawa 161 Louis Pasteur St. Ottawa ON K1N 6N5 Canada

## Abstract

Porous asymmetric polyetherimide (PEI) hollow fiber membranes with various non-solvent additives, *e.g.* lithium chloride, methanol and phosphoric acid (PA) were prepared for CO_2_ absorption and stripping process in a membrane contractor. The PEI membranes were characterized *via* gas permeation, liquid entry pressure of water (LEPw), contact angle and field emission scanning electronic microscopy analysis. The CO_2_ absorption and stripping performance was evaluated *via* the membrane contactor system. Addition of non-solvent additives increased the LEPw and membrane porosity of the PEI membrane with the formation of various membrane microstructures and contact angles. Absorption test was performed at 40 °C showed that the PEI–PA membrane produced the highest absorption flux of 2.7 × 10^−2^ mol m^−2^ s^−1^ at 0.85 m s^−1^ of liquid velocity. Further testing on PEI–PA membrane was conducted on CO_2_ stripping at 60 °C, 70 °C to 80 °C and the results indicated that the stripping flux was lower compared to the absorption flux. Stripping tests at 80 °C produced the highest stripping flux which might due to the increase in equilibrium partial pressure of CO_2_ in the liquid absorbent. Modification of PEI membrane *via* incorporation of additive can enhanced the performance of a membrane contactor *via* increasing the absorption and stripping flux.

## Abstract 2

Porous asymmetric polyetherimide (PEI) hollow fiber membranes with various non-solvent additives, *e.g.* lithium chloride, methanol and phosphoric acid (PA) were prepared for a CO_2_ absorption and stripping process in a membrane contractor.

## Graphical abstract

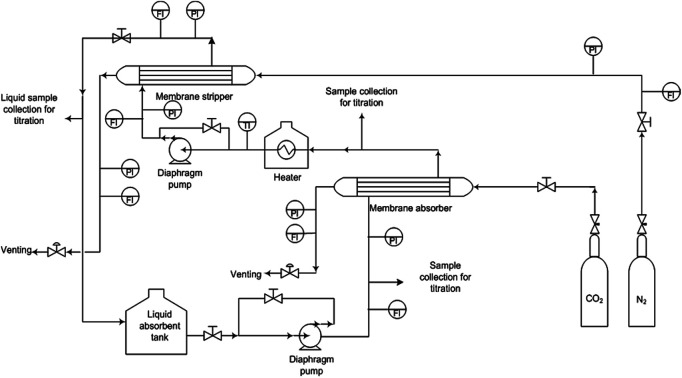

## 1. Introduction

In the conventional absorption process for CO_2_ removal, physical and chemical solvents are used extensively as liquid absorbents for the removal of acid gases especially in the petrochemical industry. Removal processing by physical solvent requires higher capital investments due to the construction cost of a high rise tower, the needs for refrigeration and the use of rotating machinery.^1^ Meanwhile, chemical solvent offers favourable advantages such as high heat of absorption and is preferable when the partial pressure of acid gas in the feed is low. Secondary amine, monoethanolamine (MEA) and diethanolamine (DEA) have been widely applied as liquid absorbents for CO_2_ removal due to their high rate of absorption. The reaction will usually lead to the formation of carbonates, bicarbonates and carbamates depending on the type of amine being used. In this case, the reaction of the secondary amine with dissolved CO_2_ is described by the zwitterions mechanism forming a carbamate ion and protonated base.^2^ Recently, the combination of membrane and amine solution which is known as a membrane contactor system has received major attention as many researchers are actively involved and have demonstrated its potential to remove CO_2_ physically or chemically *via* the absorption or stripping process.

A combination of microporous membrane and amine solution standing side by side without mixing with each other; have been a centre of attention due to its favourable features such as modular design, emulsion free, easy scaling-up, known surface area that remains undisturbed at high and low flow rates and no moving parts.^3^ This is in contrast with conventional methods which constantly creates technical problems such as flooding, entrainment and foaming. The highlight of this system is the membrane itself where it should remain non-wetted by the liquid absorbent when operated at prolong hours.

Commercial membranes such as polypropylene (PP), polytetrafluoroethylene (PTFE) and polyvinylidene fluoride (PVDF) have been diversely applied in the membrane contactor system and several experimental works have produced promising outcomes.^4–6^ Nishikawa *et al.*^4^ studied the absorption of CO_2_ from the boiler flue gases of thermal power plants by using aqueous MEA and pure water as the absorbent liquid. By operating at 50 °C and more than 275 days of operation, polyethylene (PE) and the PTFE hollow fiber membrane showed no physical deterioration due to its high degree of hydrophobicity and surface treatment with fluorocarbonic materials for the PE membrane. The overall mass transfer coefficient achieved in their study was five times larger than that of the conventional packed-bed method. Meanwhile in the stripping process, Khaisri *et al.*^6^ explored the potential of PTFE hollow fiber membrane (by Markel Corporation) for CO_2_ stripping by using aqueous MEA at 100 °C. It was found that the overall mass transfer coefficient was governed by the liquid phase mass transfer resistance and the gas phase mass transfer resistance had a minor effect on the stripping performance. This behaviour is in accordance with the performance in the absorption process by using a membrane contactor.^7,8^ Effects of membrane porosity on the long-term performance at 200 h showed that the stripping flux of the PTFE membrane with 43% porosity deteriorated significantly compared to that of a membrane with 23% porosity. Although PTFE is known as highly hydrophobic, but elevated temperature imposed during testing may be a crucial factor causing the deformation of the membrane structures; leading to continuous flux reduction.

Membrane modification by incorporating non-solvent additives such as methanol, lithium chloride and phosphoric acid have been actively demonstrated and reported in open literature specifically in the CO_2_ absorption and stripping *via* membrane contactor.^9–11^ Mansourizadeh *et al.*^9^ described the addition of glycerol, polyethylene glycol, ethanol and phosphoric acid (PA) into the PVDF polymer dope for CO_2_ absorption in distilled water. The absorption flux obtained for all membranes with different additives has a similar range with the highest absorption flux of 7.5 × 10^−4^ mol m^−2^ s^−1^ recorded for PVDF–glycerol membrane. Since the driving force for physical gas absorption is the concentration gradient, low CO_2_ absorption capacity is expected compared to chemical absorption by an amine solution. Upon addition of the additives, reduction of contact angle values was confirmed but the liquid entry pressure of the membranes was increased in comparison with plain PVDF membranes.

A previous study by Bakeri *et al.*^10^ implemented various non-solvent additives such as methanol, ethanol, glycerol and acetic acid in polyetherimide (PEI) hollow fiber membrane for CO_2_ absorption in distilled water. They highlighted that the highest absorption flux achieved was 1.85 × 10^−3^ mol m^−2^ s^−1^ for PEI–methanol which produced the lowest membrane mass transfer resistance. A similar study was focused on a PEI hollow fiber membrane for CO_2_ stripping in a DEA solution^12^ where the polymer concentration was varied and it was found that the higher concentration of polymer exhibited a higher stripping flux. Since high membrane hydrophobicity is preferred in membrane contactor applications, Zhang and Wang^13^ modified the PEI polymer by using the sol–gel method to produce organic–inorganic composite hollow fiber membranes. Their works have successfully increased the contact angle value of the pristine membrane from 80° to 120° and the composite membrane was able to withstand a long-term stability test of 30 days. However, 20% reduction of the initial CO_2_ absorption flux was reported when sodium taurinate was used as the liquid absorbent.

In this study, we focused on the performance of a PEI hollow fiber membrane for absorption and stripping in a membrane contactor system with the presence of non-solvent additives in the polymer dope. The non-solvent additives (lithium chloride, methanol and phosphoric acid) were chosen in this study due to their ability to provide narrow pore size distribution, porous network sponge-like structure and can significantly improve the existing membrane performance.^11^ The membrane properties were examined, and the CO_2_ absorption and stripping performance was further evaluated based on the specific operating condition in the membrane contactor system.

## 2. Experimental

### 2.1 Materials

Polyetherimide (PEI, Ultem) purchased from General Electric Company was used as the base polymer. *N*-Methyl-2-pyrrolidione (NMP) with a purity of more than 99.5% was purchased from Merck and used as a solvent without further purification. Methanol with a concentration of 99.9% and 99% of *n*-hexane from Merck were used for membrane post-treatment. Tap water was used as the coagulation bath medium for the spinning process. 99% MEA purchased from Merck was used to prepare 1 M aqueous solution as the liquid absorbent and 99% CO_2_ was used as the loading gas.

### 2.2 Fabrication of microporous hollow fiber membrane

Polymer concentration of 15 wt% and 4 wt% of additives (lithium chloride, methanol and phosphoric acid) were used to fabricate the hollow fiber membrane. Prior to the dope preparation, the solid polymer was dried in a vacuum oven over 24 h at 60 °C to remove moisture content. The spinning dope was prepared through homogeneous stirring of the mixture in the temperature range of 70 °C for several hours. The dopes were then degassed to remove air bubbles. Details of the spinning process can be found elsewhere.^14^ The spun fibers were immersed in water for a few days in order to ensure complete removal of the solvent. The fibers were then post-treated with methanol and *n*-hexane to minimize fiber shrinkage before drying at room temperature. Spinning conditions and parameters applied in this work are given in Table 1.

**Table 1:** PEI hollow fiber spinning conditions

Bore flow rate (ml min^−1^)	2.0
Dope extrusion rate (ml min^−1^)	4.2
Bore fluid composition	100% distilled water
Coagulation medium	Tap water
Spinneret OD/ID (mm mm^−1^)	1.15/0.55
Air gap distance (cm)	0.5
Spinning dope temperature (°C)	25
External coagulation temperature (°C)	25

### 2.3 Field emission scanning electron microscopy (FESEM) analysis

The hollow fiber samples were tested using field emission scanning electronic microscopy (FESEM; ZEIZZ SUPRA 35VP) as a standard method to analyze the morphology of the membranes. The fibers were fractured in liquid nitrogen prior to the gold coating. The images of the cross-sectional structures, as well as the outer and inner skin layers of the spun fibers were taken at various magnifications.

### 2.4 Gas permeation measurement

By using the gas permeation test, mean pore size and effective surface porosity of the outer skin layer of asymmetric membrane can be obtained.^15^ It was assumed that the pores are cylindrical and straight with gas flowing through the pores under Knudsen and Poiseuille flow regimes. Based on the common and reliable gas permeation method by Wang *et al.*,^16^ gas permeance *J*_G_ for porous membrane can be expressed as:1
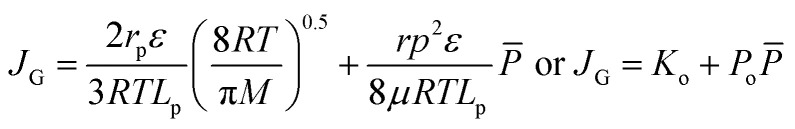
where *J*_G_ is the gas permeance (mol m^−2^ s^−1^ Pa^−1^), *r*_p_ and *L*_p_ are pore radius and effective pore length (m), respectively, *ε* is the surface porosity, *R* is gas constant 8.314 (J mol^−1^ K^−1^), *μ* is the gas viscosity (kg m^−1^ s^−1^), *M* is the gas molecular weight (kg mol^−1^), *T* is the gas temperature (K) and *P̄* is the mean pressure (Pa). Feed gas (helium) was used as tested gas for permeation experiments and the permeation flux was measured in various pressure intervals from 50 kPa to 200 kPa. The test module contained three hollow fiber membranes of 10 cm length.

The gas permeation rate was measured at room temperature using a soap bubble flow meter. Gas permeance was calculated considering the outer diameter of the hollow fiber. By plotting the gas permeance *vs.* mean pressure, *J*_G_*vs. P̄*, the intercept and slope are given as (*K*_o_) and (*P*_o_). The mean pore size and effective surface porosity can be calculated from the following equations:2
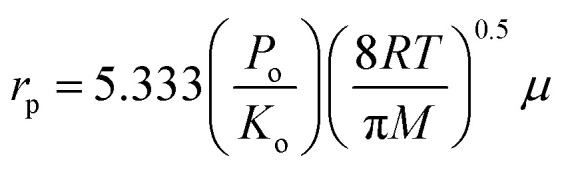
3
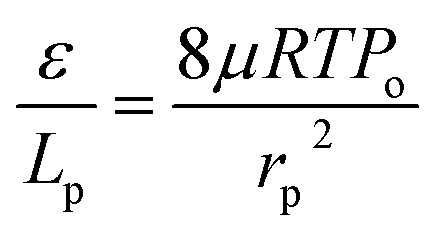


### 2.5 Liquid entry pressure and contact angle

Liquid entry pressure of water (LEPw) of each membrane was recorded when the first water droplet appeared on the outer surface of the membrane fiber. A test module consisting of one fiber was used where water was fed into the lumen of the fiber using a diaphragm pump. The pressure was slowly increased at 50 kPa intervals and kept at a constant rate at each pressure interval for about 10 min to check for the appearance of water droplets on the outer surface of the hollow fiber membrane. The fibers were tested using distilled water at ambient temperature. For water contact angle measurements, the hollow fiber was dried in a vacuum oven at 60 °C for 12 h. Meanwhile, a sessile drop technique using a goniometer (Model G1, Krauss GmbH, Germany) was used to measure the contact angle of the outer surface of the fiber. Fifteen contact angle values were measured at various positions of the sample and the average result was reported.

### 2.6 Porosity measurement

Porosity of the membrane is defined as the volume of the pores divided by the total volume of the membrane. For determination of the overall porosity, five hollow fibers with the length of 30 cm were dried for 2 h at 105 °C in a vacuum oven and weighed. The overall membrane porosity *ε*_m_ was determined by using density measurements:^16^4
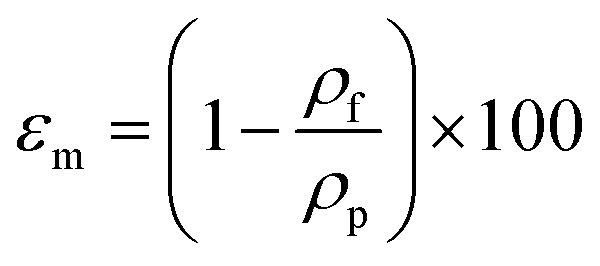
where *ρ*_f_ and *ρ*_p_ are the fiber density and polymer density, respectively. The fiber density was calculated based on the mass and volume ratio as:5
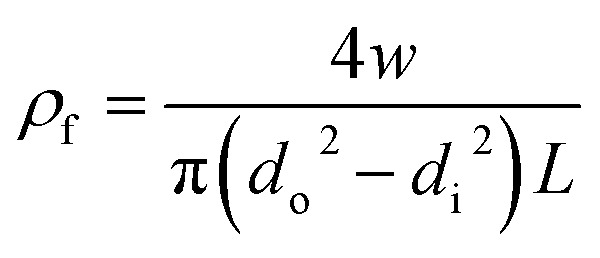
where *w* is fiber mass, *d*_o_ and d_i_ are outer and inner diameter and *L* is fiber length, respectively. The density of PEI polymer is 1.27 g cm^−3^.

### 2.7 CO_2_ absorption and stripping test

**Fig. 1:**
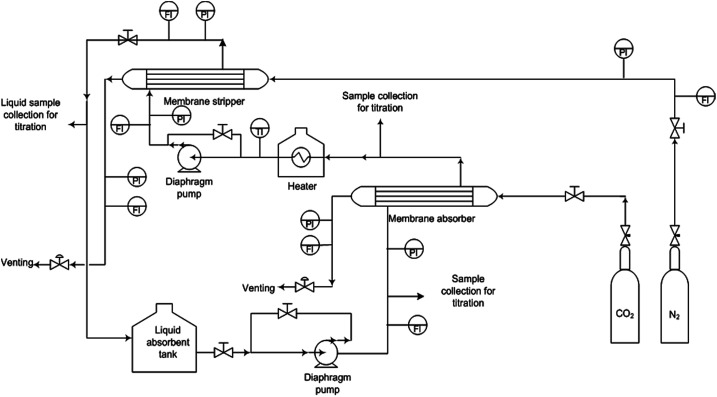
Schematic diagram of absorption and stripping test *via* membrane contactor.

Stainless steel membrane contactor modules consist of hollow fibre membranes were prepared to determine the absorption and stripping performance. Ten hollow fiber membranes were randomly installed in the module with detailed specifications as listed in Table 2. The absorption test was performed at 40 °C with 1 M of aqueous monoethanolamine (MEA) which was used as a liquid absorbent. The absorption test started by allowing the liquid absorbent to flow into the lumen and the CO_2_ gas on the shell side. The pressure difference of both phases was maintained at 50 kPa to avoid membrane wetting. The system was left to run for about 30 minutes to ascertain a steady state condition. Then, the inlet and outlet liquid were collected. The CO_2_ absorption flux was measured using chemical titration method. For the stripping test, CO_2_ loading of the system was measured according to the procedure described elsewhere.^12^ Nitrogen gas was used as sweep gas and supplied through the shell side while the absorbent liquid was flowing on the lumen side. A counter-current flow mode was applied for the gas and liquid phases. The pressure and flow rate of the gas and liquid phases were controlled by the control valves; pressure difference of 50 kPa was applied on the liquid and gas phase to avoid formation of bubbles on the liquid side. The operating temperature of 60 °C, 70 °C and 80 °C was applied during the experimental stripping test. By using the double chemical titration method,^17^ the amount of CO_2_ concentration in the inlet and outlet of the stripper module was measured to determine the stripping flux. The CO_2_ absorption and stripping flux of the membrane contactor module can be calculated as:6
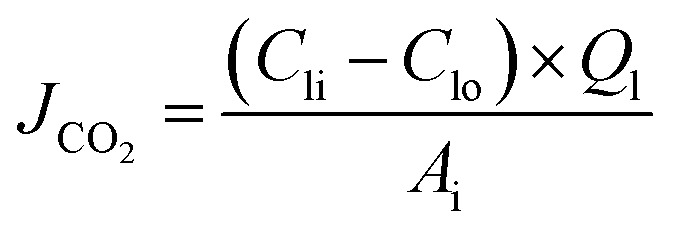
where *J*_CO_2__ is the CO_2_ absorption and stripping flux (mol m^−2^ s^−1^); *Q*_l_ is the liquid flow rate (m^3^ s^−1^); and *A*_i_ is the inner surface of the hollow fiber membranes (m^2^). The schematic diagram of absorption and stripping test is illustrated in Fig. 1.

**Table 2:** Specification of gas–liquid membrane contactor system

Module length (mm)	240
Module inner dia. (mm)	10
Fiber outer dia. (μm)	845 ± 5.3
Fiber inner dia. (μm)	565 ± 2.6
Effective fiber length (mm)	160
Number of fibers	10

## 3. Results and discussion

### 3.1 Morphology of PEI hollow fiber membranes with various additives

**Fig. 2:**
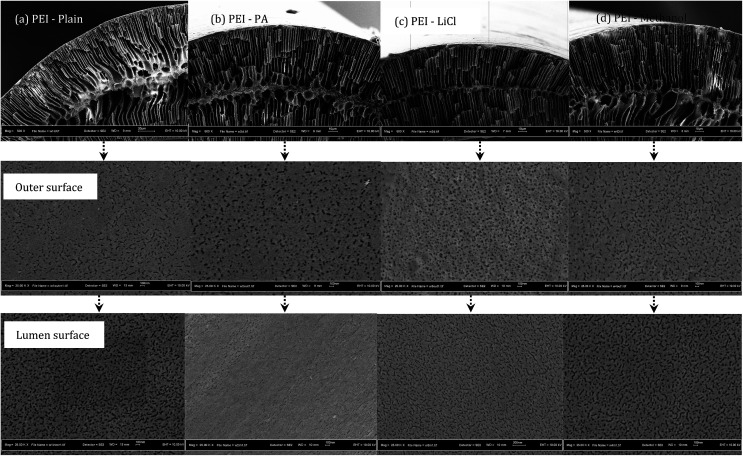
FESEM micrograph of PEI hollow fiber membrane.

The microstructure of PEI hollow fibre membranes (with and without additives) was shown in Fig. 2. All the membranes showed finger-like structure stretching from the outer surface to the inner membrane with the formation of a sponge-like layer in the middle of fibres cross section. Since water was used as the internal and external coagulant, the fast phase inversion process aids the arrangement of fine finger-like at both the inner and outer surface. It can be observed that with the presence of additives in the polymer dope system, the thick sponge-like layer which was formed in between the finger-like structure (Fig. 2a) was significantly reduced (Fig. 2b–d). This can be associated with the interaction between the polymer, additives and solvent. Upon addition of additives, the microstructure of the membrane showed depletion of macrovoid formation except for the PEI–methanol membrane. The finger-like structure stretching from the outer membrane surface to the inner membrane surface was separated by a layer of sponge like formation at the centre of the membrane cross section.

The macrovoid formation was suppressed when phosphoric acid and lithium chloride was added in the solution dope, but a reverse effect was observed for methanol. Although the morphology of all membrane samples is almost similar by the formation of thin finger-like morphology, a sponge layer exists in the middle of the fiber cross section somehow indicates a different thickness from each other. It is noted that the longer finger-like structure was formed due to rapid liquid–liquid demixing which can be attributed to the strong diffusion tendency between the water coagulation bath and solvent–non-solvent additives. The formation of the sponge-like structure in the middle of the cross-section can be correlated to its high solution viscosity (kinetic effects) that has predominant effects over the thermodynamic demixing enhancement which in turn resulted in slow solvent mutual diffusion in the polymer solution and water coagulation bath. In the presence of non-solvent additives, the instantaneous demixing mechanism could be favoured in a resulting reduced sponge-like layer. It is highly noted that with the addition of lithium chloride into the PEI in NMP dope solution, viscosity increases linearly. Both phosphoric acid and lithium chloride are ionic materials which could easily diffuse in a water coagulation bath resulting lesser microvoid formations. However, in the case of methanol as a non-solvent additive, viscosity of the dope solution is higher resulting with more microvoid formations compared to the phosphoric acid and lithium chloride non-solvent additives.

### 3.2 Characterization results of PEI membrane with different additives

Characterization results of PEI hollow fiber membranes were showed in Table 3. From Table 3, it was summarized that the addition of non-solvent additives in the polymer dope has multiple effects on the membrane properties. An increase in gas permeation was observed for PEI–PA and PEI–methanol membranes but pose an inverse effect for the PEI–LiCl membrane. This can be correlated to the low effective surface porosity that may hinder gas penetration through the membrane cross section. Other membrane properties such as liquid entry pressure demonstrated an increasing trend upon the addition of additives in the polymer dope. This increment can be associated with the development of a sponge-like structure at the middle of membrane's cross section. In addition, the formation of a different structure can be related to the rate of interaction between the polymer–solvent–additive in the phase inversion process.

**Table 3:** Characterization results of hollow fiber PEI membranes

Membranes	Gas permeance (in cm^3^ cm^−2^ s^−1^ cm Hg)	Liquid entry pressure (10^5^ Pa)	Effective surface porosity	Mean pore size (μm)	Contact angle (°)	Overall membrane porosity
PEI	0.9 ± 0.3	3.0 ± 0.3	0.39 ± 0.2	1.08 ± 0.2	76.6 ± 1.0	0.79 ± 2.0
PEI–PA	4.9 ± 0.2	5.0 ± 0.4	2.24 ± 0.5	1.14 ± 0.1	70.9 ± 0.9	0.81 ± 0.1
PEI–methanol	2.2 ± 0.4	3.5 ± 02	3.31 ± 0.3	0.45 ± 0.2	76.5 ± 1.0	0.81 ± 0.3
PEI–LiCl	0.6 ± 01	4.0 ± 0.1	0.05 ± 0.6	3.56 ± 0.3	83.4 ± 1.1	0.80 ± 0.4

### 3.3 CO_2_ absorption and stripping performance of PEI membrane in membrane contactor

**Fig. 3:**
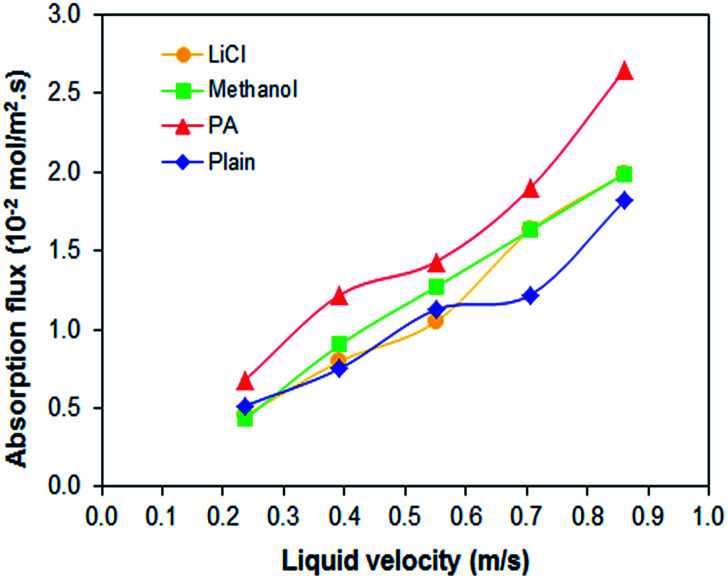
CO_2_ absorption results of PEI hollow fiber membranes with different nonsolvent additives at 40 °C.

**Fig. 4:**
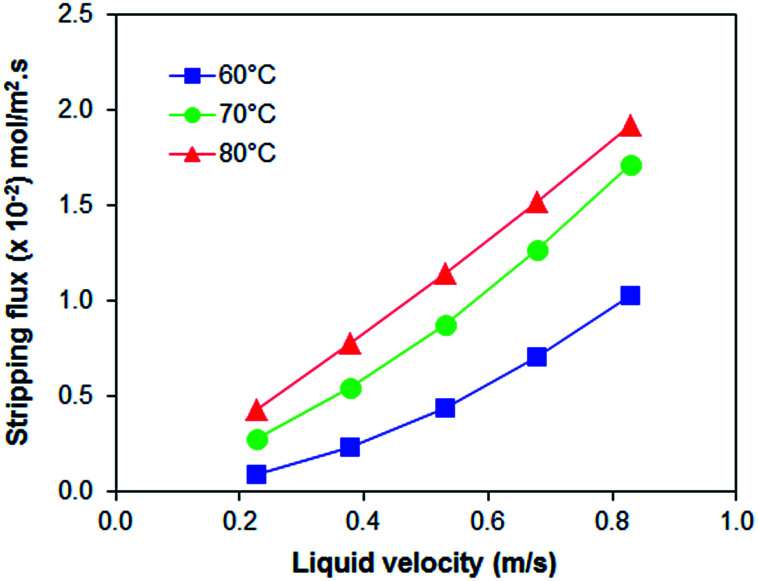
Stripping results of PEI–PA membrane at different temperature.

PEI hollow fiber membranes (with and without additives) was further evaluated for the CO_2_ absorption process at 40 °C and for the stripping process at 60 °C, 70 °C to 80 °C. As observed in Fig. 3, the absorption flux of all PEI membranes is increasing in accordance to liquid velocity increment; with PEI–PA membrane recorded the highest absorption flux. The membrane with the highest absorption flux was further evaluated for stripping test at different regeneration temperatures. It was found that at elevated temperature of 80 °C the PEI–PA membrane exhibited a higher stripping flux; as shown in Fig. 4. This can be associated with the increase in chemical reaction equilibrium constant with temperature which reduced the chemical reaction equilibrium constant; thus, leading to enhancement of the driving force to strip CO_2_ from liquid absorbent (aqueous MEA). From Fig. 4, it was noticed that the absorption flux for PEI–PA hollow fiber membrane is much higher than the stripping flux. This is due to the possible loss of CO_2_ during the stripping process through water vaporization when liquid absorbent was heated up to 80 °C. The loss of CO_2_ through vaporisation is unavoidable as the liquid absorbent used in aqueous; which can be associated with the breaking off physically bonded CO_2_–H_2_O at an elevated temperature thus leaving the chemically bonded CO_2_–MEA to remain in the solution. To achieve a high stripping flux, the absorption flux of CO_2_ in the amine solution should be high and these can be done by increasing the solution concentration and stripping temperatures. However, both parameters have its own limitation that might intensify the corrosion effect towards the module and contactor system when operated at longer operation and increasing temperature. In the industrial sector, the stripping process uses conventional liquid amine usually performed at temperature exceeding 100 °C. However, when using the membrane contactor system (amine coupled with membrane), the range of stripping temperature should be considered especially when some of the polymeric membrane are unable to endure the striping operation at very high temperatures. Therefore, further investigation on the membrane properties by various modification methods should be highlighted to ensure that the existing polymeric membrane can tolerate high-temperature operations.

### 3.4 Comparison with literature

Membrane technology processes specially for absorption and stripping methods *via* membrane contactor system have showed promising impact in term of lower energy consumption and seen as a viable alternative for industrial application. Recently, much study has focused on membrane contactor with aqueous amine absorbent for CO_2_ absorption and stripping. However, combination on both process; absorption and stripping are scarcely reported elsewhere in the literature. Enormous studies on absorption by using distilled water for CO_2_ removal have been reported elsewhere.^18–20^ In real operation, utilising distilled water as liquid absorbent to remove CO_2_ may cause several problems such as physical loss of the water content due to higher operating temperature for stripping process and pore forming. Rongwong *et al.*^21^ performed an absorption test by using various liquid absorbent include water, MEA, DEA, and AMP. The result showed that absorption flux increased in order of MEA > AMP > DEA > water. It can be concluded from their study that chemical absorbents were more reactive towards CO_2_ compared to water. Therefore, employing chemical solvent is more practical then distilled water specially in industrial application.

The structure of the fabricated membrane also has significant impact on the absorption and stripping performances. Kianfar *et al.*,^22^ conducted a research to investigate the structure of the membrane on high performance of absorption and stripping processes. It showed that by addition of 2 wt% ethanol into dope polymer solution would formed porous membrane compared to membrane without ethanol addition. Meanwhile, the absorption and stripping fluxes increased as the liquid velocity increased. The highest absorption flux of 3.9 × 10^−3^ mol m^−2^ s^−1^ and stripping flux of 2.00 × 10^−4^ mol m^−2^ s^−1^ was achieved at liquid flow rate 300 and 200 ml min^−1^, respectively.

Amine solvents are the most common liquid absorbent used by researchers because of high CO_2_ loading capacity, high absorption rate and low cost for regeneration. MEA is one the amine group solvent which known as very good solvent and most preferable in many industries. Some interesting result had been observed by researchers by studying the performance comparison of ammonia and MEA as liquid absorbent for CO_2_ absorption. Cui and deMontigny^23^ investigated the used of ammonia as liquid absorbent in comparison with MEA. Comparison study showed that the CO_2_ absorption using MEA as liquid absorbent was much higher compared to ammonia. It can be related to the less reactivity properties of ammonia compared to MEA. Table 4 summarizes several findings of absorption and stripping performances have been reported by various researchers for the past 10 years.

Research findings on CO_2_ absorption and stripping flux by other researchers^a^

**Table:** 

CO_2_ absorption				
Researcher	Year	Membrane type	Liquid absorbent	CO_2_ absorption flux (mol m^−2^ s^−1^)
Rajabzadeh *et al.*^24^	2009	HF PVDF	1 M MEA	8.0 × 10^−3^
			2 M MEA	1.25 × 10^−2^
		HF PTFE	3 M MEA	1.4 × 10^−2^
			4 M MEA	1.4 × 10^−2^
Marzouk *et al.*^5^	2010	PTFE	5 M MEA	2.03 × 10^−3^
			5 M DEA	1.86 × 10^−3^
			5 M TETA	2.12 × 10^−3^
Chen *et al.*^25^	2011	PTFE	0.03 AMP	1.8 × 10^−4^
			0.03–0.06 MEA	
			0.015 M PZ	
Lv *et al.*^26^	2012	PP	0.5 M MEA	4.4 × 10^4^
Ghasem *et al.*^27^	2012	PVDF/triacetin	0.5 M NaOH	(1–3.2) × 10^−3^
Franco *et al.*^28^	2012	Plasma treated-PP	MEA	Plasma treated-PP > untreated-PP
Rajabzadeh *et al.*^29^	2013	PVDF, PTFE	2 M MEA	1.8 × 10^−2^
Mansourizadeh and Mousavian^30^	2013	PVDF–glycerol	DEA	0.03
McLeod *et al.*^31^	2014	PP	Ammonia	2.3 × 10^−4^
Chabanon *et al.*^32^	2014	PTFE, PP, PVDF, nylon	MEA	Not reported
Rongwong *et al.*^33^	2015	PTFE	MEA	PTFE > PVDF using MEA liquid
		PVDF	AMP	
Rahim *et al.*^34^	2015	PVDF	Amino acid solution	Not reported
Hashemifard *et al.*^18^	2015	PDMS coated PEI	Distilled water	7.29 × 10^−4^
Rezaei Dasht Arzhandi *et al.*^19^	2015	PEI + 1 wt% MMT	Distilled water	2.2 × 10^−3^
		PVDF + 5 wt% MMT		1.9 × 10^−3^
Sadoogh *et al.*^20^	2015	PVDF	1 M MEA	7.2 × 10^−4^
			1 M DEA	6.5 × 10^−4^

a HF = hollow fiber, FS = flat sheet, PVDF = polyvinylidene fluoride, PP = polypropylene, PTFE = polytetrafluoroethylene, PEI = polyetherimide, MEA = monoethanolamine, DEA = diethanolamine, TETA = triethylenetetramine, PZ = piperazine, K_2_CO_3_ = potassium carbonate, PEG = polyethylene glycol, MMT = montmorillonite.

**Table:** 

CO_2_ stripping				
Researcher	Year	Membrane type	Liquid absorbent	CO_2_ stripping flux (mol m^−2^ s^−1^)
Koonaphapdeelert *et al.*^35^	2009	Ceramic HF	MEA	Not reported
Khaisri *et al.*^6^	2011	PTFE	3 M, 5 M, 7 M MEA	3 M = 7.5 × 10^−4^
				5 M = 13.5 × 10^−4^
				7 M = 13.0 × 10^−4^
Simioni *et al.*^36^	2011	FS PTFE	K_2_CO_3_	1.97 × 10^−2^
		PALL		1.31 × 10^−2^
Naim *et al.*^11^	2012	HF PVDF	1 M DEA	1.61 × 10^−2^
Naim *et al.*^37^	2012	HF PVDF/PEG	1 M DEA	4.03 × 10^−2^
Naim *et al.*^38^	2013	HF PVDF	1 M DEA	1.5 × 10^−2^
Naim and Ismail^39^	2013	HF PEI	1 M DEA	2.7 × 10^−2^
R. Sisakht *et al.*^40^	2013	HF PVDF	1 M DEA	3.0 × 10^−4^
Naim *et al.*^41^	2014	HF PVDF	DEA	4.0 × 10^−2^
		HF PEI		3.5 × 10^−2^
Tarsa *et al.*^42^	2015	PEI	0.1 M MEA	5.1 × 10^−4^
Kianfar *et al.*^22^	2017	HF PSF	1 M MEA	2.00 × 10^−4^

## 4. Conclusion

Polyetherimide (PEI) hollow fiber membranes with additives have been tested for CO_2_ absorption and stripping process in a membrane contactor system. Multiple effects have been observed from the characterization test which includes the increase in membrane porosity and wetting pressure. Morphology study of the membrane cross section showed that the combination of a finger-like and sponge-like structure of PEI–phosphoric acid (PA) aid to the high gas permeation and high wetting pressure. Further evaluation of the PEI–PA membrane performance in membrane contactor was executed for the absorption and stripping process. The membrane showed the highest absorption flux and was further evaluated for the stripping test. It was observed that at a higher stripping temperature of 80 °C, the membranes could obtain the highest stripping flux compared at lower temperatures. It is expected that at a further increment of temperature beyond 80 °C would produce a higher stripping flux but somehow will sacrifice the system equipment to corrosion problems in the long run.

## Conflicts of interest

There are no conflicts to declare.

## Supplementary Material
